# Optical laser systems at the Linac Coherent Light Source

**DOI:** 10.1107/S1600577515006244

**Published:** 2015-04-22

**Authors:** Michael P. Minitti, Joseph S. Robinson, Ryan N. Coffee, Steve Edstrom, Sasha Gilevich, James M. Glownia, Eduardo Granados, Philippe Hering, Matthias C. Hoffmann, Alan Miahnahri, Despina Milathianaki, Wayne Polzin, Daniel Ratner, Franz Tavella, Sharon Vetter, Marc Welch, William E. White, Alan R. Fry

**Affiliations:** a Linac Coherent Light Source, SLAC National Accelerator Laboratory, 2575 Sand Hill Road, Menlo Park, CA 94025, USA

**Keywords:** FEL, ultrafast lasers, pump–probe

## Abstract

Ultrafast optical lasers play an essential role in exploiting the unique capabilities of recently commissioned X-ray free-electron laser facilities such as the Linac Coherent Light Source (LCLS). Pump–probe experimental techniques reveal ultrafast dynamics in atomic and molecular processes and reveal new insights in chemistry, biology, material science and high-energy-density physics. This manuscript describes the laser systems and experimental methods that enable cutting-edge optical laser/X-ray pump–probe experiments to be performed at LCLS.

## Abstract 2

This manuscript serves as a reference to describe the optical laser sources and capabilities at the Linac Coherent Light Source.

## 1. Introduction   

X-ray free-electron lasers (FELs) provide unprecedented brightness and time resolution, fundamentally changing the landscape of X-ray research in dynamic phenomena at the atomic, molecular and nanostructural scales. Approximately 75% of all experiments at the Linac Coherent Light Source (LCLS) require optical laser excitation to initiate a reaction or state within the sample under investigation. The vast majority of the physical phenomena under investigation by pump–probe techniques occur on a timescale of picoseconds to femtoseconds, requiring both optical and X-ray sources to have femtosecond pulse duration and a well defined and controllable temporal separation. Typically the optical pulse is used to excite (pump) a sample into a transient state prior to interrogation (probe) by an X-ray pulse. In certain experiments the X-ray pulse is used as the pump and the optical pulse is used as the probe; regardless, both the pump and probe and their relative timing are of fundamental importance in all such experiments. LCLS and other developing X-ray FEL facilities have made significant investments in equipment and expert personnel to support a robust and evolving capability for ultrafast optical laser sources. These capabilities have been driven by the user community, and have dramatically influenced the scientific capabilities and productivity of these facilities. This article reviews the current state of optical laser system parameters available to the users of LCLS and describes novel optical sources available to the LCLS user community including terahertz (THz; 10^12^ Hz), mid-infrared (mid-IR) and sub-10 fs sources.

## 2. Laser systems overview   

### 2.1. Near Experimental Hall optical beamlines   

**Figure 1:**
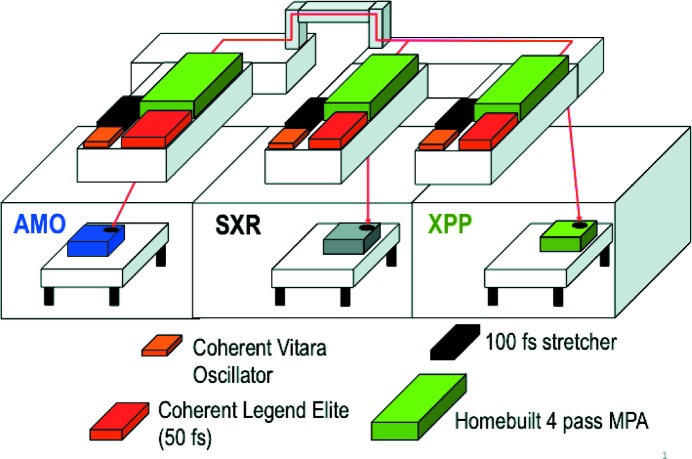
Schematic showing the general layout of the NEH Laser Hall atop the first three experimental hutches at LCLS. The optical lasers within the Laser Hall are transported well over 10 m to the hutch laser enclosures.

Ultrafast optical lasers are integrated into each instrument in LCLS. In the Near Experimental Hall (NEH), the lasers are located one floor above the main experimental floor, directly above the X-ray beamlines (Fig. 1 ▶). There is a laser associated with each of the three experimental hutches, that is, one each for the Atomic, Molecular and Optical (AMO) (Ferguson *et al.*, 2015 ▶), Soft X-ray (SXR) (Dakovski *et al.*, 2015 ▶) and X-ray Pump–Probe (XPP) (Chollet *et al.*, 2015 ▶) hutches. These systems are almost identical, beginning with a Ti:sapphire oscillator front-end (Vitara, Coherent Inc.) capable of producing ultrashort pulses that are synchronized to the master clock of LCLS. The oscillators were modified to run at a repetition rate of 68 MHz, matching the seventh sub-harmonic of the 476 MHz frequency used for electron beam production and acceleration. The cavity length can be changed *via* a stepper motor and a piezo actuator for precise phase control (see §3.2). The Vitara is capable of producing seed bandwidths from 30 nm to 125 nm FWHM with a central wavelength near 800 nm.

The first amplification stage in our systems is a commercially available chirped-pulse amplification (CPA) regenerative amplifier (Legend Elite USP, Coherent Inc.). Prior to amplification, the ultrashort oscillator pulse is stretched *via* a dispersive grating stretcher. Depending on the experiment or application, the amount of stretching the seed pulse undergoes can be varied. Normally, we use a 1200 lines mm^−1^ stretcher grating on the full bandwidth pulse produced from the seeding laser. Here, the geometry of the stretcher limits the effective seeded bandwidth to 30–40 nm FWHM. For applications requiring narrower bandwidths, longer pulse durations or lower peak powers, a pair of spectral filters is added to an optional 1800 lines mm^−1^ grating stretcher line to limit the seeded bandwidth to 7–10 nm FWHM. If no further amplification of the laser is required, the lower-energy regenerative amplifier beam may be transported to the experimental hutches. Compression of the stretched pulse occurs within the experimental hutch using a corresponding single- or dual-grating compressor matching the stretcher in the laser hall. The typical transmission efficiency of the compressor system is about 70%. For pulses with the full bandwidth of 30 nm, a transform limit, a 30 fs (FWHM) pulse is produced while pulses containing 7–10 nm of bandwidth will result in transform-limited pulse durations of 100 to 150 fs (FWHM).

**Figure 2:**
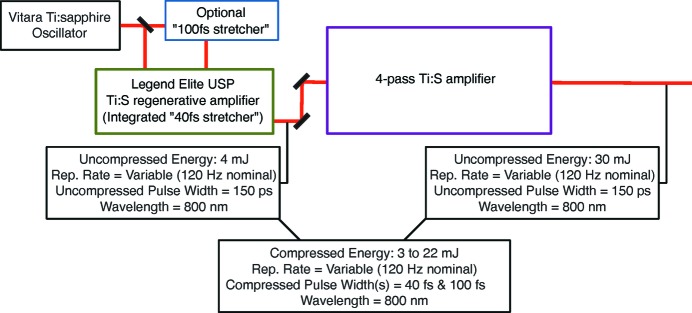
Box diagram of the layout for the core optical laser systems at LCLS.

Frequently, LCLS experiments require optical pulse energies greater than 3 mJ of compressed energy. When higher pulse energies are needed, a second stage of amplification may be implemented. Each of the three main experimental laser systems in the NEH Laser Hall have a custom-built four-pass amplifier (multi-pass), capable of boosting the nominal regenerative amplifier beam to 30 mJ, with 0.2% r.m.s. energy stability, at 120 Hz repetition rate. The multi-pass amplifier can be used with the broad- or narrow-bandwidth seed pulses it receives from the regenerative amplifier front end. Refer to Fig. 2 ▶ for typical 800 nm optical parameters.

### 2.2. Far Experimental Hall optical beamlines   

Unlike for the NEH, the lasers deployed in most of the Far Experimental Hall (FEH) experimental instruments [Coherent X-ray Imaging (CXI) (Liang *et al.*, 2015 ▶) and Matter in Extreme Conditions (MEC) (Nagler *et al.*, 2015 ▶)] reside within the hutches themselves. X-ray Coherent Scattering (XCS) (Alonso-Mori *et al.*, 2015 ▶) has not had a laser system; in 2015, however, XCS will be expanded to create a dedicated laser room adjacent to the experimental area to house a laser system capable of providing almost identical optical parameters (800 nm, 40 fs, 5 mJ, 120 Hz) to those of the regenerative Ti:sapphire amplifiers described in §2.1.

The CXI instrument is capable of performing experiments requiring moderate optical laser parameters. CXI’s standard configuration is identical to that of the 4 mJ, 800 nm, Ti:sapphire regenerative amplifier systems that make up the front-end for all of the NEH experimental laser platforms. In some circumstances CXI can offer the possibility to amplify the pulse energy to almost 25 mJ by way of a portable, home-built, multi-pass amplifier pumped by a flash-lamp pumped Nd:YAG laser running at 120 Hz.

The MEC instrument houses the most unique laser capabilities at LCLS which are described in the companion article in this special issue (Nagler *et al.*, 2015 ▶).

### 2.3. Enhanced laser capabilities   

Experiments at the LCLS often require non-standard laser parameters, including a wide range of wavelengths, pulse energies, pulse durations and specialized optical configurations. This section describes some of the unique laser capabilities provided for user experiments.

#### 2.3.1. Visible/UV OPA   

Many experiments require optical laser excitation that falls outside of the harmonics of a Ti:sapphire laser (800 nm, 400 nm, 267 nm and 200 nm). In these cases, an Optical Parametric Amplifier (OPA) is used to fill many of the gaps between the 800 nm fundamental and harmonics. A list of typical wavelengths and pulse energies is given in Table 1 ▶.

#### 2.3.2. Narrowband mid-IR   

The operating wavelength range of the OPA can be extended past 2400 nm by difference frequency generation (DFG). Here the signal and idler outputs of the OPA are mixed in a nonlinear crystal, such as gallium selenide (GaSe) and silver gallium selenide (AgGaSe_2_), to produce mid-infrared (MIR) radiation. The resulting output ranges in wavelength from 4 to 20 µm. Several users have requested relatively narrow bandwidths in this regime, typically a FWHM bandwidth of less than 15% of the central wavelength. For example, if the central wavelength of the MIR pulse is 15 µm, typical FWHM bandwidths are of the order of 1.7 to 2 µm.

LCLS has developed a portable all-in-one optical table assembly solely for the purpose of generating narrow-band MIR laser pulses. To minimize the spectral bandwidth of the generated MIR, a transform limit, 120–150 fs, 800 nm pump drives a commercially available high-energy optical parametric amplifier (HE-TOPAS, Light Conversion Ltd). The portable table has a suite of MIR diagnostics [HgCdTe (MCT) detectors, quadrant detectors and scanning spectrometer] to fully characterize the divergence, pulse energy and spectrum and has been successfully deployed in all of the NEH experimental hutches for user experiments (Först *et al.*, 2014 ▶; Ferguson *et al.*, 2015 ▶; Dakovski *et al.*, 2015 ▶; Chollet *et al.*, 2015 ▶).

#### 2.3.3. Laser-based terahertz sources   

In addition to traditional mid- and far-infrared wavelengths described in the previous section, LCLS also provides users with intense single-cycle terahertz (THz) pulses. These broadband pulses cover the THz range of the electromagnetic spectrum (1 THz 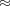 300 µm 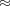 33 cm^−1^ 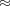 4.1 meV) and can be obtained by optical rectification of femtosecond laser pulses. The resulting pulses are inherently carrier envelope stable and their electric field can be mapped out in the time domain. While THz pulses in free space have been available for more than 25 years (Fattinger & Grischkowsky, 1989 ▶), their energies have typically been far too small to be used as a pump in ultrafast experiments.

The tilted-pulse-front method (Hebling *et al.*, 2002 ▶) has been used to generate intense THz single-cycle pulses with energies of tens of microJoules from optical rectification in LiNbO_3_ (Yeh *et al.*, 2007 ▶), opening the field of nonlinear THz spectroscopy. The spectrum of the resulting THz pulses extends to about 3 THz and is usually peaked between 0.4 and 1 THz.

Alternatively, optical rectification can be performed in organic salts with exceptionally high nonlinear coefficients, pumped with near-infrared (NIR) femtosecond pulses (Schneider *et al.*, 2006 ▶; Hauri *et al.*, 2011 ▶). These crystals, such as DAST or DSTMS, have low absorption in the wavelength range between 1200 and 1500 nm and allow for collinear phase matching for THz frequencies. The NIR pump wavelengths are generated with a multi-stage OPA, providing pulse energies of up to 3 mJ in the NIR when pumped with 20 mJ, 100 fs, 800 nm pulses. In this case, the peak spectral amplitude is centered at 2 THz with frequency components extending to 5 THz or more, depending on the duration of the NIR pump pulse. With these techniques, pulse energies up to 15 µJ and peak field strengths approaching 1 MV cm^−1^ can be reached at the sample location.

Single-cycle THz pulses have successfully been used at LCLS to resonantly excite collective excitations in multiferroics (Kubacka *et al.*, 2014 ▶), for X-ray pulse characterization by THz streaking (Grguras *et al.*, 2012 ▶) or field-induced switching of correlated electron systems. Since most of these applications require the sample to be in an ultra-high-vacuum environment, great care must be taken to guide the THz excitation pulse efficiently into the diffraction chamber. Interested readers are encouraged to read the article by Turner *et al.* (2015) ▶ for a detailed description of the implementation of the soft X-ray THz instrument.

#### 2.3.4. Sub-10 fs pulses   

In experiments using optical lasers, the temporal resolution of the measurement is governed by three factors: the laser pulse duration, the X-ray pulse duration and the temporal jitter between these two pulses. With cross-correlation techniques measuring pump–probe arrival times down to the few femtosecond level and the demonstration of X-ray pulse durations of sub-10 fs, the limitation on temporal resolution is the ∼50 fs duration of the optical laser pulses. A factor of five reduction in the laser pulse duration to below 10 fs would result in a similar factor of improvement in the temporal resolution achievable in these experiments.

Reduction of the laser pulse duration can be achieved using hollow fiber pulse compression, in which the existing laser pulse bandwidth is broadened through interaction with a gaseous medium and subsequently compressed to a shorter duration. Hollow fiber pulse compression has been successfully deployed using the hutch laser system described in §2.1 to generate sub-10 fs pulses with pulse energies up to 500 µJ energy per pulse. Pulses as short as 7.4 fs have been measured. Future plans for this capability include shorter pulses, higher-energy pulses, and a mobile setup that can be deployed to all experimental endstations.

#### 2.3.5. Pulse-to-pulse polarization control   

Femtosecond pulses at 800 nm can also be delivered with two alternating polarization states. Examples include experiments studying all optical magnetic switching where the magnetic state can be controlled by left- or right-handed circular polarized light (de Jong *et al.*, 2013 ▶). This can be achieved by using a combination of Pockels cells and wave plates. The Pockels cell drivers can be triggered in an arbitrary pulse pattern. Because of potential damage of the Pockels cells the pulse energies at 800 nm are limited to less than 100 µJ.

## 3. Integration of optical laser systems in X-ray experiments   

In all experiments employing some type of pump–probe technique the success of the experiments is critically dependent on precisely defined spatial and temporal overlap between the optical pulse and the X-ray pulse.

### 3.1. Spatial overlap   

Depending on the instrument or experimental endstation, several options can be deployed to establish spatial overlap in the sample interaction region. Direct viewing of the X-ray and laser beams is the simplest means by which spatial overlap is achieved. Typically the location of the X-ray beam is determined from the visible fluorescence produced when the X-rays impinge on a scintillator such as a cerium-doped yttrium aluminium garnet (Ce:YAG) screen or painted-on phosphor that is placed in the same plane as the experimental sample. Visible and near-infrared wavelengths generate fluorescence in these screens, and the focal spot can simply be positioned in the same location as the X-ray spot. Fluorescent tape or heat-sensitive liquid crystal sheets have also been used successfully to determine the spatial location of the laser pulses, particularly in the mid-infrared.

In some cases it is not possible to view the beams directly, such as when using MIR or THz radiation. In these cases a pinhole can be used to determine the X-ray location, initially by maximizing the transmission of the X-rays through the pinhole, and subsequently by optimizing the laser beam position to maximize the laser transmission. The fraction of laser light transmitted can also give some indication of the focal spot size. Similarly, knife-edge measurements of the spatial profile can be used to determine the center and width of both the laser and X-ray spots. The laser beam can then be positioned so that the center of the beam coincides with the center of the X-ray spot.

In all cases, care must be taken to ensure that the spatial overlap is performed in the sample plane. This is especially true when the laser and X-rays are non-collinear, as the beams will walk-off in the propagation direction. For example, in the modest case of 50 µm focal spots and a 10° angle between the beams, the spots will completely walk-off in <300 µm.

### 3.2. Timing and synchronization   

Taking full advantage of the temporal resolution of femtosecond X-ray pulses and femtosecond optical lasers in pump–probe experiments requires timing measurement and control of the variable delay between the pulses on a timescale of a few 10 fs or better. LCLS operates at a maximum repetition rate of 120 Hz and has a pulse-to-pulse timing jitter relative to the accelerator radio-frequency (RF) distribution of approximately 60 fs RMS, integrated over a bandwidth of 0.1–100 kHz. Optical lasers must be locked to the accelerator RF distribution with similar or better timing jitter, and drifts in the laser beam path and RF distribution need to be controlled to approximately the 1 ps level. At the time of writing, the original fiber-based LCLS laser timing system (Byrd *et al.*, 2010 ▶) is being replaced with an RF-based system described in this section.

X-ray pulse timing is determined by the electron beam timing, which is in turn controlled by the RF fields in the accelerator cavities. The accelerator RF sources are locked to a 476 MHz RF coaxial cable phase distribution system. This ‘RF reference’ is re-stabilized to the electron beam with each shot, using electron arrival time measured at RF phase cavities in the undulator hall. The RF reference is then transmitted through a stabilized coaxial cable to the experimental hutches, where it is used as a phase reference for the laser locking systems described in this text.

In each laser system, the mode-locked seed laser oscillator operates at 68 MHz, the seventh sub-harmonic of the 476 MHz RF reference frequency, which defines the fine (sub-picosecond) timing of laser pulses in the absence of configuration changes to the laser system.

The noise performance of the LCLS laser locking system was measured to have an RMS laser-to-RF-reference timing jitter of 25 fs between 100 Hz and 100 kHz. Below 100 Hz, phase noise is dominated by the noise of the linac RF reference. Drift of the RF-based locking system has been measured at less than 1 ps day^−1^, but can be higher during times of large external temperature variation. This is higher drift compared with the previous fiber-based system, and future upgrades to the LCLS laser locking system will include drift correction, possibly using a parallel fiber distribution system. A detailed description of the timing distribution and oscillator locking electronics developed at SLAC is given by Gumerlock *et al.* (2014) ▶.

To surpass the limitations of this jitter, experiments involving the fastest physical phenomena (*e.g.* transitions of core shell electrons) measure the relative timing between the laser and X-rays on a shot-by-shot basis by means of X-ray/optical cross-correlation techniques described in §3.3.

### 3.3. Temporal overlap and time-of-arrival measurement   

Rough timing down to the sub-10 ps level between the laser and X-rays is often achieved by inserting a device (MSM detector or SMA wire or connector) with a fast time response for both the X-rays and optical laser into the interaction region. The waveforms from the devices are then measured on a remotely controlled oscilloscope, typically with a bandwidth exceeding 10 GHz. However, these time scales are typically too large to observe meaningful ultrafast phenomena. More precise timing (<100 fs) can be found using a variety of techniques depending on the X-ray wavelengths and sample environments. For almost all X-ray wavelengths at LCLS, a silicon nitride (Si_3_N_4_) membrane or bismuth (1,1,1) crystal can be inserted in the sample plane (Fritz *et al.*, 2007 ▶). A downstream camera or diode is then used to monitor the transmission (or reflection) of the optical laser through (or off) the target material. As the laser delay time is stepped from laser before X-rays to X-rays before laser, the optical transmission (or reflection) is altered, revealing temporal overlap.

Timing at the timescale of the optical laser pulses and X-ray pulses, *e.g.* 10 fs or less, requires a quantified measure of the relative timing on a shot-to-shot basis. In order to account for uncorrelated sources of shot-to-shot timing jitter and drift, LCLS has developed cross-correlation techniques to measure the relative time of arrival between the X-ray pulse and the optical laser pulses. With this relative timing information, each data point in each LCLS experiment is re-sorted with every recorded data point re-binned according to the measured delay between the X-rays and the optical laser.

Numerous cross-correlation techniques have been developed at LCLS and other facilities, all relying on X-ray-induced material changes that cause prompt (a few femtoseconds) changes in the optical properties of the material (typically silicon nitride), with the ability to resolve the relative timing within a time window in the range of order a few picoseconds. These techniques include changes in the reflectivity of a thin membrane illuminated by a laser pulse at a 45° angle (Schorb *et al.*, 2012 ▶) or changes in the transmission and reflection of a membrane illuminated by a temporally chirped laser pulse (Bionta *et al.*, 2011 ▶). Both methods have measured temporal accuracy of the order of 25 fs RMS, and both methods are currently in operation at LCLS.

In addition to the above-mentioned techniques, an approach has been developed that combines both X-ray-gated spatially and spectrally encoded techniques (Hartmann *et al.*, 2014 ▶). In this configuration the X-ray and optical beams are crossed in a silicon nitride membrane and their relative delay is encoded in the spatial beam profile of the optical probe. The resulting temporally encoded optical pulse is sent into a spectrometer to produce a two-dimensional spectrogram, similar to that in the optical technique of frequency-resolved optical gating (FROG). The two-dimensional spectrogram produced with this technique provides an over-sampled data-set that is less susceptible to X-ray mode variations. The resulting temporal accuracy can be better than 1 fs RMS with sufficient X-ray fluence on the cross-correlator.

## 4. Conclusion   

Ultrafast optical lasers play an essential role in the study of ultrafast dynamics in X-ray FEL experiments by providing precisely timed, wavelength tunable, variable-energy laser pulses to samples, creating precisely controlled dynamic conditions in diverse materials relevant to chemistry, material science, molecular biology and high-energy-density physics. Significant developments in the ultrafast optical capabilities are anticipated in the near future ranging from self-evident scaling in terms of pulse energy, pulse duration and extended wavelengths to novel developments such as spectral phase programming of optical pulses for coherent control of transient states, high-energy and high-repetition-rate lasers for producing states of high pressure and high energy density, and for excitation and probing at the <1 fs time scale.

## Figures and Tables

**Table 1:** Pulse energies available in the near-infrared and visible spectrum Nonlinear frequency conversion is performed in an OPA and subsequent mixer stages. Values in this table are for an OPA pumped by 2.5mJ pulses at 800nm, 45fs and 120Hz repetition rate. P: Pump, S: Signal, I: Idler, SH: second harmonic, FH: fourth harmonic, SF: sum frequency.

Wavelength (nm)	Process	Pulse energy (J)
11502400	(S + I)	550
575800	(SHS)	80
8001150	(SHI)	50
475532	(P + S, SFS)	75
533600	(P + I, SFI)	80
290400	(FHS)	12
400480	(FHS)	8
